# Deep supervision and atrous inception-based U-Net combining CRF for automatic liver segmentation from CT

**DOI:** 10.1038/s41598-022-21562-0

**Published:** 2022-10-10

**Authors:** Peiqing Lv, Jinke Wang, Xiangyang Zhang, Changfa Shi

**Affiliations:** 1 Department of Software Engineering, Harbin University of Science and Technology, Rongcheng, 264300 China; 2 School of Automation, Harbin University of Science and Technology, Harbin, 150080 China; 3 Mobile E-business Collaborative Innovation Center of Hunan Province, Hunan University of Technology and Business, Changsha, 410205 China

**Keywords:** Biomedical engineering, Three-dimensional imaging, Tomography, Image processing, Machine learning

## Abstract

Due to low contrast and the blurred boundary between liver tissue and neighboring organs sharing similar intensity values, the problem of liver segmentation from CT images has not yet achieved satisfactory performance and remains a challenge. To alleviate these problems, we introduce deep supervision (DS) and atrous inception (AI) technologies with conditional random field (CRF) and propose three major improvements that are experimentally shown to have substantive and practical value. First, we replace the encoder's standard convolution with the residual block. Residual blocks can increase the depth of the network. Second, we provide an AI module to connect the encoder and decoder. AI allows us to obtain multi-scale features. Third, we incorporate the DS mechanism into the decoder. This helps to make full use of information of the shallow layers. In addition, we employ the Tversky loss function to balance the segmented and non-segmented regions and perform further refinement with a dense CRF. Finally, we extensively validate the proposed method on three public databases: LiTS17, 3DIRCADb, and SLiver07. Compared to the state-of-the-art methods, the proposed method achieved increased segmentation accuracy for the livers with low contrast and the fuzzy boundary between liver tissue and neighboring organs and is, therefore, more suited for automatic segmentation of these livers.

## Introduction

Accurate liver segmentation is essential in liver cancer diagnosis and surgical planning. In traditional clinics, radiologists still manually decline liver segmentation from CT in a slice-by-slice fashion, which is labor-intensive and prone to errors due to observer dependence. Therefore, automatic liver segmentation is highly desirable and valuable in real-time clinical applications. However, its accuracy has not achieved satisfactory performance. It remains a challenge due to low contrast and the fuzzy boundary between liver tissue, the neighboring organs sharing similar intensity values, and severe pathology around the liver borders.

Numerous methods have been proposed for automatic liver segmentation in the past few decades, in which deep learning-based approaches have received widespread attention since they are free from hand-crafted feature engineering.

Deep learning-based methods are closely related to the rapid development of computer hardware in recent years. According to the type of neural network segmentation and its variants, they can be divided into FCN-based and U-Net-based methods. Among the many strategies proposed for semantic segmentation, FCN has received widespread attention for its novel end-to-end mode^1^. Many researchers have employed FCN to segment the liver. For example, Ben Cohen et al.^2^ used FCN for liver segmentation and liver lesion detection for the first time. Christ et al.^3,4^ proposed a double FCN cascade method to segment the liver and tumor regions and optimized the segmentation results with 3D dense CRF. Yuan et al.^5^ proposed a three cascade FCN to segment liver and tumor automatically. Han et al.^6^ designed a 24-layer FCN model, which adopted a skip connection similar to U-Net between encoder and decoder to fuse low-layer details and high-layer semantic information. Kaluva et al.^7^ suggested putting dense modules into FCN and achieved good liver and liver tumor segmentation results. Vorontsov et al.^8^ designed a residual block and long skip connection similar to ResNet to connect 21 convolutional layers for liver tumor segmentation and further reduce the number of parameters.

FCN-based methods effectively solve the problems of significant storage overhead and low segmentation efficiency of traditional CNN. However, due to the lack of correlation between global pixel information, the results obtained by FCN are not precise enough. Ronneberger et al.^9^ proposed the U-Net based on FCN and achieved tremendous success in medical image segmentation. Compared with FCN, U-Net combines low-layer and high-layer semantic information through skip connection, significantly improving segmentation accuracy. Then, enhanced U-Net models have been extensively studied. For example, Roth et al.^10^ segmented liver and liver tumors through cascaded 2D U-Net. Li et al.^11^ presented the H-DenseU-Net model, which combines 2D U-Net and 3D U-Net models to fully use the information in and between slices. Jin et al.^12^ performed a 3D hybrid residual attention-aware strategy, combining residual structure with U-Net to extract liver and tumor in CT image. Seo et al.^13^ added a residual path with deconvolution and activation operations in the skip connection of U-Net. Ansari et al.^14^ proposed a novel neural network (Res‑PAC‑UNet) that employs a fixed‑width residual UNet backbone and Pyramid Atrous Convolutions, providing a low disk utilization method for precise liver CT segmentation. And the proposed network is trained on the medical segmentation decathlon dataset using a modified surface loss function. Experimental results demonstrate that the proposed network achieves a dice coefficient of 0.950 ± 0.019 with less than half a million parameters. Gao et al.^15^ proposed ASU-Net++ based on U-Net++ and dilated convolution. It modified the original Atrous Spatial Pyramid Pooling (ASPP) into an adaptive pooling structure nested in U-Net. Their experimental results show the advantage of handling different tumor sizes with complex margins. Wang et al.^16^ proposed a SAR-U-Net network model, which combines the advantages of attention mechanism, residual and multi-scale modules, and used it to process 2D liver images. Zhang et al.^17^ proposed the SAA-Net network model, combining the benefits of Scale Attention and Axis Attention, demonstrating its effectiveness in small-sized tumor segmentation. Zhou et al.^18^ proposed an automatic liver segmentation network based on multi-scale feature fusion (MSFF-Net), which leverages 3D V-Net, residual, dilated convolution, and deep supervision. Kushnure et al. conducted extensive research on improving the U-Net framework and applied it to liver and tumor segmentation^19–22^. e.g. They^19^ presented an end-to-end trained multi-scale UNet architecture, MS-UNet, based on the Res2Net and SENet modules. Then they^20^ introduced an improved deep learning-based multi-scale UNet++ (M2UNet++), demonstrating effectiveness on the 3DIRCADb dataset. Besides, Kushnure et al.^21^ also suggested the MFCA-Net model, which leverages the advantages of Res2Net, and the attention mechanism, and demonstrated its good performance on the CHAOS dataset. Furthermore, they^22^ proposed the HFRU-Net network, which uplifted the liver and tumor segmentation performance by modifying the high-level and low-level features using feature fusion and multi-scale feature extraction techniques. They reported higher accuracy even with fuzzy boundaries between the liver and tumor. However, the performances of these segmentation approaches are still unsatisfactory in the accuracy and robustness.

The abovementioned methods work well when dealing with regular liver segmentation or certain challenge cases. However, these approaches are likely to fail when applied to the liver with low-contrast neighboring organs, mainly caused by insufficient detail learning at the boundary.

To alleviate this problem, we introduce a new network framework using DS and AI and call it DA-UNet. The contributions of the proposed DA-UNet with CRF refinement (The source code is available at https://github.com/lvpeiqing/DA-UNet-CRF) are enumerated as follows.

- The 2D convolution in the U-Net network is extended to 3D convolution to effectively use adjacent slices' spatial information. In addition, we replace the encoder's standard convolution with the residual block, which aims to increase the depth and width of the network to avoid gradient vanishment.
- Provide atrous inception (AI) module to connect the coder and decoder. Atrous inception (AI) allows us to obtain multi-scale features.
- Incorporate the deep supervision mechanism (DSM) into the decoder to improve the discrimination and robustness of early-layer learning, the gradient vanishing/explosion problem is solved, and training effectiveness is enhanced.
- Employ a fully connected conditional random field (CRF). This helps to improve the labeling deviation of adjacent data and leads to further refinement of the overall boundary details of the liver.

The other parts of the paper are organized as follows: In “Method” section describes the proposed network's architecture. Then, in “Experiments and results” section, the experiments and results are provided in detail, and the last section summarizes the research work of this paper.

## Method

### Architecture

**Figure 1:**
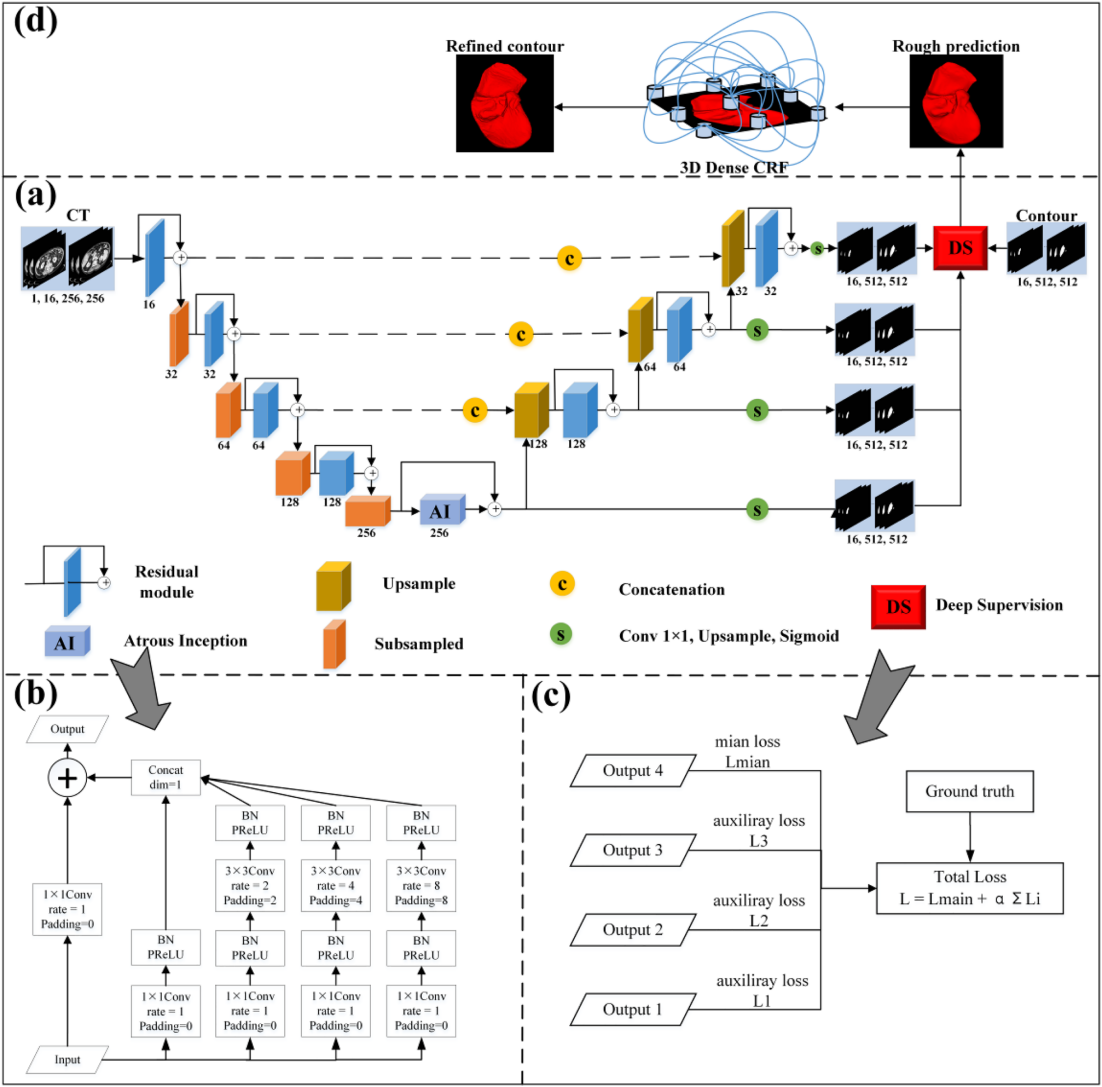
The architecture of the proposed framework. (**a**) DA-UNet (**b**) AI (**c**) DS (**d**) CRF.

The proposed network architecture is depicted in Fig. 1. The left contraction path is connected with the right expansion path through a skip connection to form a symmetrical structure. Specifically, the image features are extracted in the contraction path and located in the expansion path. The overall framework consists of eight residual blocks, four down-sampling blocks, one AI module, three up-sampling blocks, four output modules, and a DS mechanism. The size of the input and prediction images are 256 × 256 × 16 and 512 × 512 × 16, respectively. Furthermore, the proposed framework uses convolution with a 2 × 2 × 2 kernel instead of the maximum pooling layer, and all the convolution operations use 3 × 3 × 3 filters. Besides, we employ the parameterized rectification linear unit (PReLU) as the nonlinear activation function.

### AI module

We replace the convolution operation of U-Net with the residual module to obtain a deeper network and overcome the gradient vanishing problem. Specifically, we introduced the AI module as the bridge layer to connect the encoder and the decoder. The proposed module draws on the idea of the inception^23^, and we integrate the atrous convolution^24^ with dilation rates of 2, 4, and 8 into the 3 × 3 convolution (Fig. 1b), to maximize the capture of different scales of contextual information, and finally merge.

**Figure 2:**
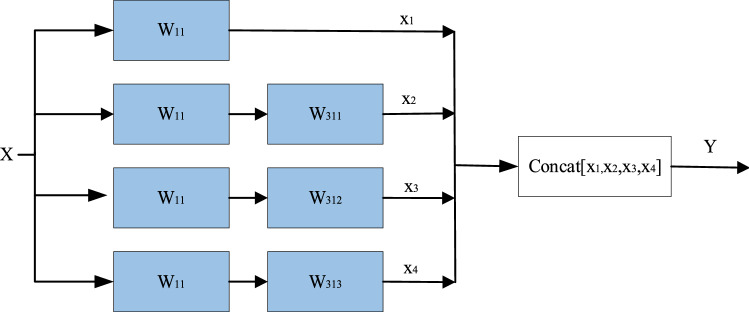
A detailed description of the AI module uses a 3 × 3 convolution kernel with dilated convolutions of 2, 4, and 8 to extract features, and the extracted features are merged with concat operation.

The main details of the AI module are shown in Fig. 2. *X* represents the input feature, *W*_11_ represents a convolution with a convolution kernel of 1 × 1, a rate of 1, and a padding of 0. *W*311, *W*312, and *W*313 represent a 3 × 3 convolution with expansion rates of 2, 4, and 8, respectively. *x*_1_, *x*_2_, *x*_3_, and *x*_4_ represent the features obtained by convolution kernels of different sizes. *Y* represents the output of the AI module. *Y* is calculated as follows:

1 $${x}_{1}={w}_{11}x+{b}_{11}$$

2 $${x}_{2}={{w}_{311}(w}_{11}x+{b}_{11})+{b}_{311}$$

3 $${x}_{3}={{w}_{312}(w}_{11}x+{b}_{11})+{b}_{312}$$

4 $${x}_{4}={{w}_{313}(w}_{11}x+{b}_{11})+{b}_{313}$$

5 $$Y={w}_{y}(Concat[{x}_{1},{x}_{2},{x}_{3,}{x}_{4}])+{b}_{f}$$

### DS mechanism

We employed the DS^25^ in the decoder (Fig. 1c). At each decoding branch stage, first, point convolution is used to process the input feature map. Then, trilinear interpolation is utilized for up-sampling. Finally, the Sigmoid layer calculates the probability map of segmentation results and compares them with the corresponding labels. Thus, the loss of the intermediate and the final output layers are combined for gradient backpropagation to effectively reduce the influence of gradient vanishment, improve learning efficiency and accelerate the optimization. Equation (6) gives the loss function of each iteration of the DS as follows,

6 $$L={L}_{main}+\alpha \sum_{i=1}^{N}{L}_{i}(i=\mathrm{1,2},3)$$

where *L*_*main*_ is the primary loss function, representing the loss value output by the final decoder, and *L*_1_, *L*_2_, and *L*_3_ are defined as auxiliary loss functions, meaning the loss value output by the intermediate decoder, respectively. The parameter α is the deep supervision coefficient. Since the deep layers usually contain more complex features than the shallow layers, we can improve the segmentation accuracy by assigning a higher weight to the loss of the deep network output. In this paper, parameter α is updated every 40 epochs according to the formula α = 0.8 × α. The training of the proposed model and all other models used for comparison in the paper is demonstrated in Algorithm 1.
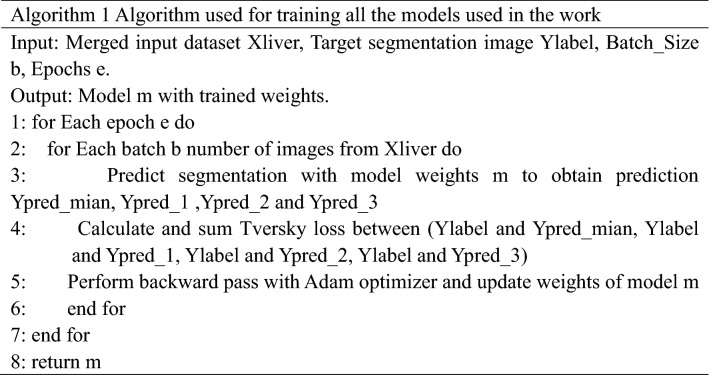


### Loss function

Since the number of background pixels accounts for most of the CT image, it may cause severe data imbalance. Milletari et al.^26^ proposed the Dice coefficient score (DSC) loss function to alleviate this problem. However, Dice loss is the average value of precision and recall, which weighs False Positive (FP) and False Negative (FN) equally. Then, Salehi et al.^27^ took the difference between FP and FN into account and proposed a Tversky loss function based on the Tversky similarity index; that is, by continuously adjusting these two hyperparameters *α* and *β*, it performs a trade-off between FP and FN to achieve the optimal effect. Therefore, we used the Tversky-based similarity index as the final loss function in this paper, which is defined as follows:

7 $$T(\alpha ,\beta ) = 1 - \frac{{\sum\nolimits_{i = 0}^{L} {\sum\nolimits_{j = 0}^{N} {p_{ij} g_{ij} } } }}{{\sum\nolimits_{i = 0}^{L} {\sum\nolimits_{j = 0}^{N} {p_{ij} g_{ij} + \alpha \sum\nolimits_{i = 0}^{L} {\sum\nolimits_{j = 0}^{N} {\left( {p_{ij} g_{ij}^{\sim} } \right)^{2} } } + \beta \sum\nolimits_{i = 0}^{L} {\sum\nolimits_{j = 0}^{N} {\left( {p_{ij}^{\sim} g_{ij} } \right)^{2} } } } } }}$$

where *i* represents the ground truth, *j* represents the index of pixels in the image, *L* represents the total number of classes in the segmentation task, and *n* represents the total number of pixels. We use *p*_*ij*_ to represent the probability that the pixel *j* belongs to class *i* during prediction and *g*_*ij*_ to represent the probability that pixel *j* belongs to class *i* in the ground truth. If pixel *j* of the input image belongs to category 0, then, *g*_0*j*_ = 1, and *g*_1*j*_, *g*_2*j*_…, *g*_*ij*_ = 0. In addition, two parameters, *α* and *β,* are adjusted for the ratio between FPs and FNs. The sum of *α* and *β* is equal to 1. Specifically, when *α* = *β* = 0.5, the Tversky loss function is equivalent to the Dice loss function.

### Evaluation metrics

In this paper, five volume and surface-based metrics are used for evaluation^28,29^, including Dice, volume overlap error (*VOE*), relative volume error (*RVD*), average symmetrical surface distance (*ASD*), and root mean square symmetrical surface distance (*RMSD*). Dice and ASD are the two most critical indicators for liver segmentation evaluation. Assuming that, A is the segmentation result of the liver and B is the ground truth, then the definitions of the five metrics are as follows:

1. Dice: the similarity of two sets whose range is [0,1]. The larger the value, the higher the segmentation accuracy.
  8 $$Dice(A,B) = \frac{{2\left| {A \cap B} \right|}}{\left| A \right| + \left| B \right|}$$
2. Volume Overlap Error (VOE): the difference between the segmented volume and the ground truth volume.
  9 $$VOE(A,B) = 1 - \frac{{\left| {A \cap B} \right|}}{{\left| {A \cup B} \right|}}$$
3. Relative Volume Error (RVD): Used to determine whether the segmentation result is over-segmented or under-segmented. The closer the value is to zero, the higher accuracy of the segmentation.
  10 $$RVD(A,B) = \frac{\left| B \right| - \left| A \right|}{{\left| A \right|}}$$
4. Average Symmetric Surface Distance (ASD): the average distance between the surfaces of segmentation results *A* and the ground truth *B*, where *d* (*v*, *S*(*X*)) represents the shortest Euler distance from voxel *v* to the surface voxel of the segmentation result.
  11 $$ASD(A,B) = \sqrt {\frac{1}{{\left| {S(A)} \right| + \left| {S(B)} \right|}}} \left( {\sum\limits_{{p \in S(A)}} {d(p,S(B)) + } \sum\limits_{{q \in S(B)}} {d(q,S(A)} )} \right)$$
5. Root Mean Square Symmetric Surface Distance (RMSD): the maximum surface distance between the segmentation results *A* and the ground truth *B*.
  12 $$RMSD(A,B) = \sqrt {\frac{1}{{\left| {S(A)} \right| + \left| {S(B)} \right|}}} \sqrt {\sum\limits_{p \in S(A)} {d^{2} (p,S(B)) + } \sum\limits_{q \in S(B)} {d^{2} (q,S(A))} }$$

### Ethical approval

This article does not contain any studies with live human participants or animals performed by any of the authors.

## Experiments and results

### Datasets

In the experiment, we use three publicly available datasets, including LiTS17, 3DIRCADb, and SLiver07, acquired by a wide variety of CT scanners from different vendors (The three datasets are publicly available at https://competitions.codalab.org/competitions/17094, https://www.ircad.fr/research/3d-ircadb-01/, and https://sliver07.grand-challenge.org/). We outline their specifications in Table 1, and we confirm that all experiments were performed in accordance with relevant guidelines and regulations.

**Table 1:** The specifications of the experimental datasets (“–” means none).

Datasets	Training	Test	Size	In-plane resolution (mm)	Inter-slice resolution (mm)	Slice num
LiTS17	130	70	512 × 512	0.55–1.0	0.45–6.0	42–1026
3DIRCADb	20	–	512 × 512	0.56–0.81	1.25–4	74–225
SLiver07	20	10	512 × 512	0.56–0.8	1–3	64–394

Considering that LiTS17 and SLiver07 do not provide the golden standards for the test sets, we randomly divided LiTS17 and Sliver07 training datasets into new training sets and new test sets according to 116/15 and 10/10, respectively. In addition, since 3DIRCADb does not provide a test set, its 20 datasets with golden standards are randomly divided into training and test sets according to 10/10.

### Settings

During the training, we set the entire batch to 800 and batch size to 1. The initial learning rate is set to 0.001, and the learning rate is adjusted according to the set interval number. The selection of interval numbers depends on the batch setting, and the selected batches are 350 and 650. The learning rate is updated according to the formula $lr=initial\_lr\times \gamma$; that is, when the training batch reaches 350 and 650, the learning rate begins to attenuate, where the initial value of *γ* is 0.1. We use standard Adam to optimize the objective function. All the experiments were run on a PC with Ubuntu 18.04, equipped single Intel Xeon silver 4110 CPU, RTX2080ti GPU, 64G RAM, and Pytorch1.4 as the deep learning framework.

**Figure 3:**
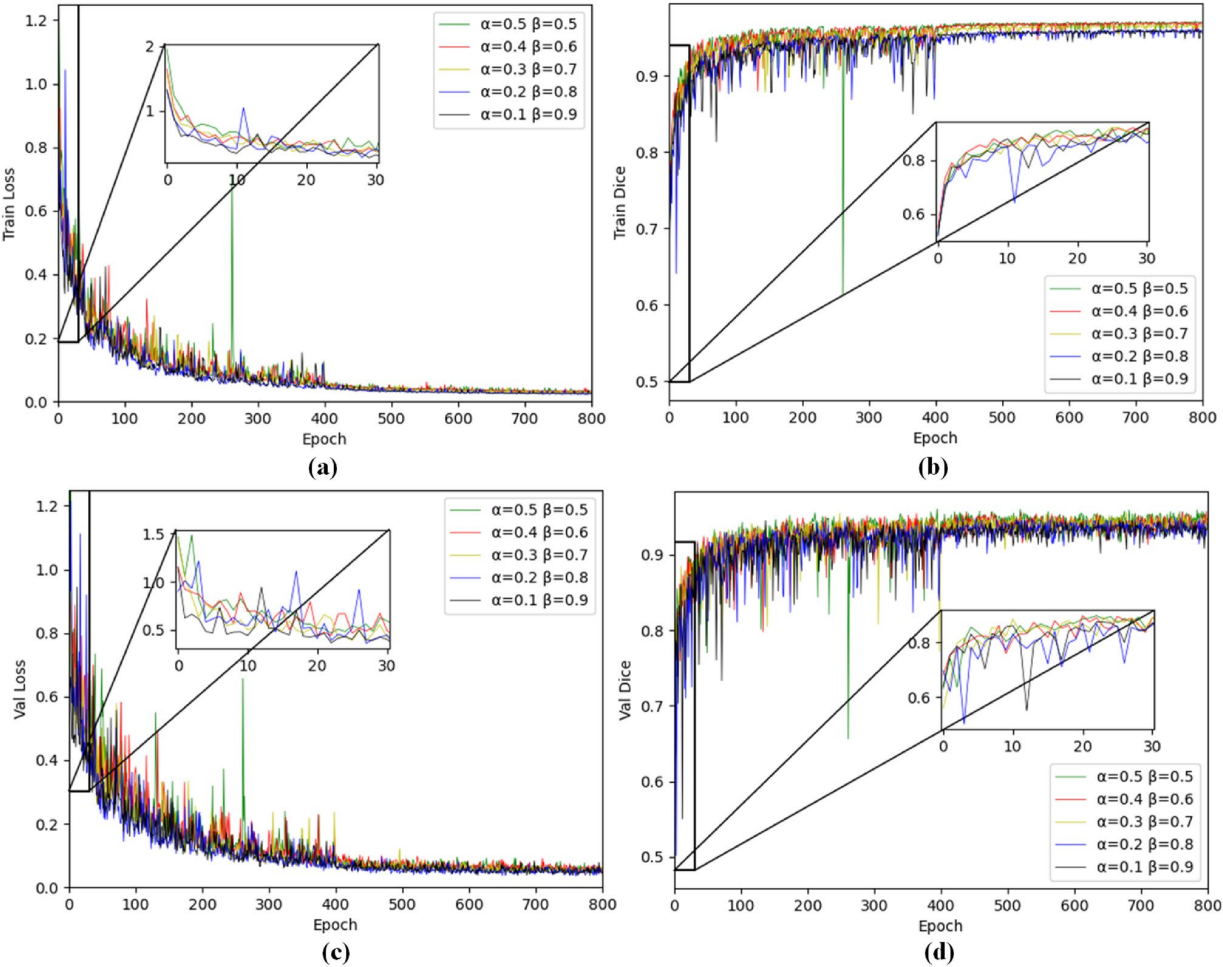
Visualization of loss and accuracy in training and validation process on LiTS17 dataset. (**a**) training loss, (**b**) training accuracy, (**c**) verification loss, and (**d**) verification accuracy.

Figure 3 shows the training and verification process of the proposed 3D DA-UNet when α and β take different values for the Tversky loss function. As can be seen from the figures, when α and β take 0.4/0.6 (red), the loss in the training and verification phases fluctuates slightly, with the highest initial value of Dice, which indicates the most conducive to avoiding the gradient vanishing/explosion problem. Therefore, we empirically set the hyperparameters to 0.4 and 0.6 in this paper.

To improve computational efficiency, we preprocess the datasets. First, we downsample each volume of the input to 256 × 256. Second, we locate the initial and final sections of the liver region and expand 20 slices outward. Finally, to exclude irrelevant organs, we unified Hounsfield intensity to[− 200, 200], set the spacing of the z-axis of all data to 1 mm, and normalized the intensity to [0,1].

### Ablations

In this section, we implement the ablation experiments on three public datasets to verify the effectiveness of the proposed model combination. A total of three combined models were adopted, including 3D U-Net (Baseline), 3D ResU-Net (+ Res), 3D AI-UNet (+ Res + AI), and 3D DA-UNet (+ Res + AI + DS). It can be seen from Table 2 that, with the superposition of the network modules, the segmentation performances are incrementally improved.

**Table 2:** Quantitative analysis results of ablation experiments on three databases.

Dataset	Model	Dice (%)	VOE (%)	RVD (%)	ASD (mm)	RMSD (mm)
LiTS17	3D U-Net	91.96 ± 0.65	7.76 ± 1.20	0.87 ± 0.28	1.58 ± 0.57	5.26 ± 4.59
	+ *CRF*	*92.63* ± *1.44*	*6.49* ± *2.63*	*0.68* ± *0.31*	*1.62* ± *1.44*	*5.09* ± *4.73*
	3D ResU-Net	94.62 ± 0.50	7.54 ± 0.93	0.68 ± 0.26	1.34 ± 0.64	4.94 ± 5.51
	+ *CRF*	*95.15* ± *0.59*	*5.54* ± *1.11*	*0.57* ± *0.16*	*1.49* ± *0.92*	*4.25* ± *5 .47*
	3D AI-UNet	95.01 ± 0.53	7.23 ± *0.89*	0.61 ± *0.29*	1.36 ± *0.71*	4.88 ± *5.12*
	+ *CRF*	*95.85* ± *0.49*	*6.51* ± *0.71*	*0.55* ± *0.21*	*1.30* ± *0.85*	*4.51* ± *3.25*
	3D DA-UNet	96.71 ± 0.45	6.37 ± 0.84	0.53 ± 0.19	1.22 ± 0.25	4.54 ± 5.00
	** + *CRF***	***97.62* ± *0.27***	***4.64* ± *0.51***	***0.42* ± *0.11***	***1.07* ± *0.49***	***2.39* ± *0.72***
3DIRCADb	3D U-Net	92.97 ± 0.65	7.73 ± 1.26	0.40 ± 0.13	3.44 ± 1.83	8.68 ± 6.88
	+ *CRF*	*94.96* ± *0.59*	*7.59* ± *1.19*	*0.39* ± *0.15*	*2.65* ± *2.02*	*8.13* ± *7.83*
	3D ResUNet	95.80 ± 0.59	7.08 ± 1.27	0.36 ± 0.23	1.45 ± 1.91	3.26 ± 1.04
	+ *CRF*	*97.10* ± *0.16*	*5.62* ± *1.16*	*0.31* ± *0.12*	*0.98* ± *1.31*	*2.68* ± *1.21*
	3D AI-UNet	95.91 ± 0.61	6.81 ± 1.32	0.32 ± 0.26	1.49 ± 1.65	3.13 ± 1.28
	+ *CRF*	96.12 ± 0.25	5.12 ± 1.12	0.29 ± 0.32	1.32 ± 1.22	2.85 ± 1.23
	3D DA-UNet	96.54 ± 0.66	6.69 ± 1.24	0.22 ± 0.47	1.34 ± 0.33	2.64 ± 0.59
	** + *CRF***	***98.17* ± *0.19***	***3.58* ± *0.38***	***0.18* ± *0.12***	***0.95* ± *1.31***	***2.57* ± *0.32***
SLiver07	3D U-Net	94.24 ± 0.57	6.11 ± 1.31	0.73 ± 0.28	3.76 ± 3.10	9.93 ± 8.14
	+ *CRF*	*94.63* ± *0.52*	*5.10* ± *1.03*	*0.71* ± *0.24*	*3.36* ± *2.01*	*8.86* ± *6.77*
	3D ResUNet	96.43 ± 0.38	4.29 ± 1.03	0.48 ± 0.26	2.02 ± 1.53	6.87 ± 5.83
	+ *CRF*	*97.14* ± *0.39*	*3.51* ± *0.85*	*0.36* ± *0.19*	*1.70* ± *0.88*	*5.56* ± *4.58*
	3D AI-UNet	97.12 ± 0.42	4.31 ± 0.99	0.42 ± 0.25	1.58 ± 1.23	5.58 ± 4.65
	+ *CRF*	97.51 ± 0.39	3.65 ± 0.78	0.39 ± 0.28	1.38 ± 0.95	4.12 ± 3.21
	3D DA-UNet	97.84 ± 0.33	4.23 ± 0.63	0.21 ± 0.35	1.09 ± 0.09	4.77 ± 5.06
	** + *CRF***	***98.68* ± *0.36***	***2.61* ± *0.51***	***0.19* ± *0.14***	***1.07* ± *0.06***	***3.40* ± *4.17***

Significant values are in bolditalics and italics.

As the residual structure was added and compared with 3D U-Net, the 3D ResU-Net achieved superior performance on the main metrics, proving the contribution of residual structure to the performance improvement. Then as the AI module was superposed, the segmentation accuracy of the 3D AI-UNet was improved. Finally, as the DS was integrated, the performance of the 3D DA-UNet was further enhanced, proving the DS's effectiveness.

Moreover, to validate the effectiveness of 3D dense CRF post-processing, we added the 3D dense CRF process based on the above ablations. It can be seen from Table 2 that, after employing 3D dense CRF, the performances of all models are improved.

**Figure 4:**
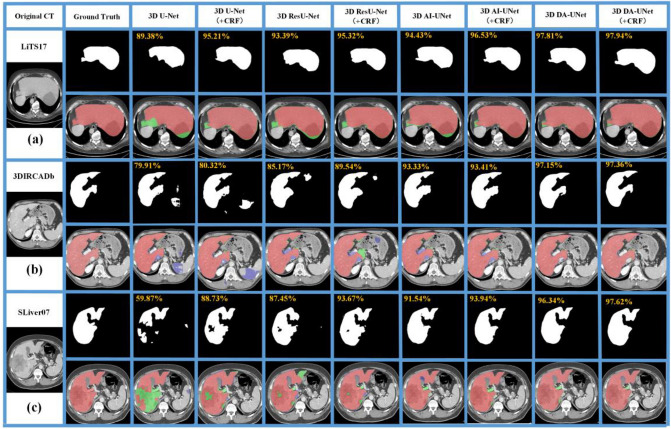
Visualization of the ablation experiments (The red region denotes the ground truth, blue/green region represents the under-/over-segmentation) (**a**) liver adjacent to other organs (**b**) liver adjacent to other organs (**c**) liver with border tumor.

Figure 4 shows some typical visual results of the ablations on three datasets. In Fig. 4a and c, 3D-Net showed severe over-segmentation errors. As 3DResU-Net is introduced, the segmentation result is significantly improved, mainly due to the employment of residual structure that makes the network deeper and wider to extract more image features. Then, after the AI module is integrated into 3DResU-Net, the fuzzy liver boundary is refined. The segmentation accuracy is improved since the AI module extracts more image features of different scales. Finally, when the DS is employed, thanks to the rationality of the top-layer output, the 3D DA-UNet further improves the segmentation accuracy, with refinements on details of some small areas. Figure 4b demonstrated a typical liver case adjacent to other organs, in which 3D U-Net shows a severe under-segmentation error. As the residual structure, AI, and the DS are integrated, the under-segmentation error is reduced continuously. Moreover, when 3D dense CRF is employed, the segmentation errors are reduced, and the result reaches the best in all cases.

**Figure 5:**
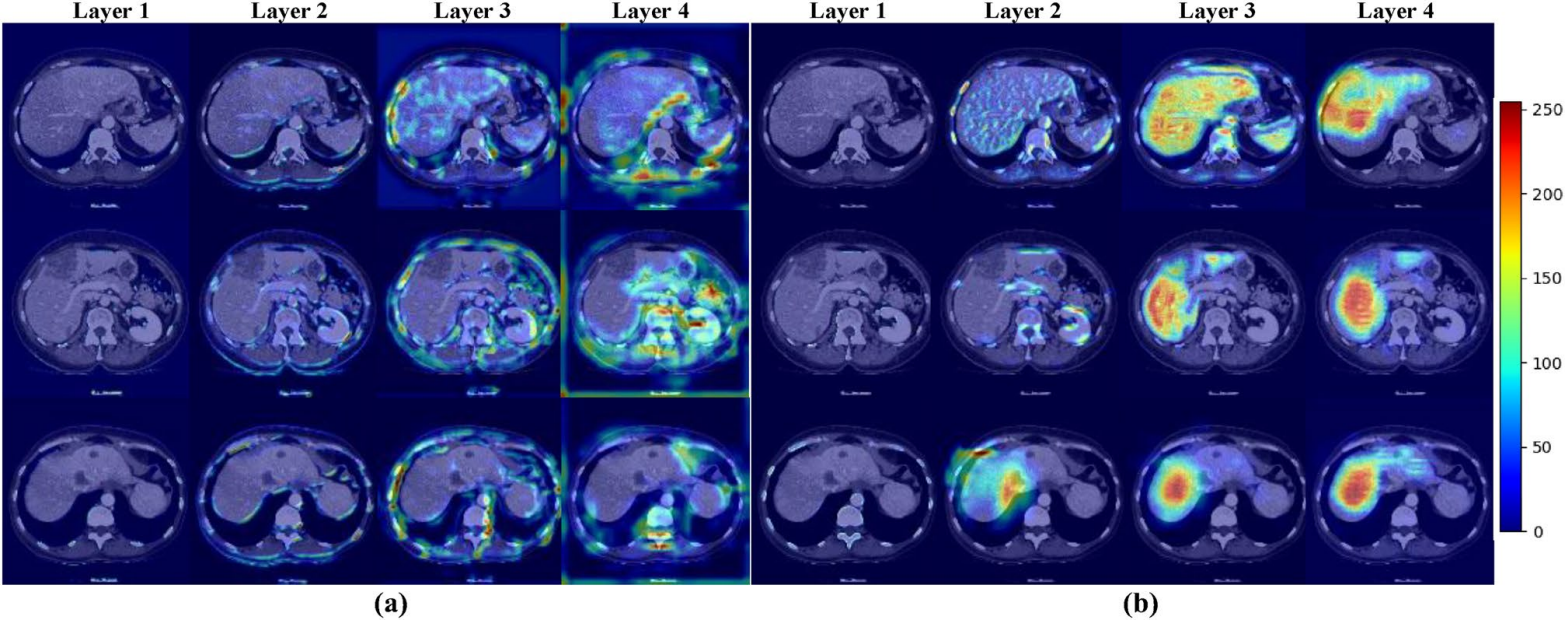
Heat maps obtained from different feature layers with and without DSM from layers 1–4. (**a**) without DSM (**b**) with DSM.

In addition, we provide the feature heat maps of the first four layers using the proposed 3D DA-UNet with and without DSM (Fig. 5), respectively. It can be seen from the figure that, without DSM (Fig. 5a), most of the image features learned by the network at the shallow layer focus on the outer contour of the liver, including the features of non-target organs (kidney, rib, spleen et al.). As the layer of the network increases, the region of interest in the model expands. However, the high heat map still focused on the boundary of the liver and other organ regions.

In contrast, with DSM (Fig. 5b), the highlight of the liver area at the shallow layer is significantly enhanced. As the layers of the network increase, the high heat area gradually approaches the ground truth, making the liver area steadily refined.

### Time-costs

Table 3 provides the training and test time of the proposed method on three different datasets. Compared with 3D U-Net, 3D ResU-Net, and 3D AI-UNet, the proposed DA-UNet method enabled a deeper and wider network while inevitably increasing the time cost of training and testing. It can be seen from the table that the proposed method takes the least training and test time. In addition, due to the complexity of the proposed method, the average training and test time without/with post-processing are slightly higher than those of 3D U-Net, 3D ResU-Net, and 3D AI-UNet. Moreover, we also found that the test time is significantly increased after employing CRF. Nevertheless, it is acceptable to trade off the best segmentation performance at a certain time cost.

**Table 3:** Training and testing time-costs of various methods on three different datasets.

Datasets	Method	Training time	Test time
LiTS17	3D U-Net	57 h 42 min 17 s	42.78 s
	3D ResU-Net	59 h 13 min 54 s	48.46 s
	3D AI-UNet	60 h 23 min 45 s	49.32 s
	**3D DA-UNet**	**61 h 28 min 12 s**	**51.76 s**
	*3D DA-UNet* + *CRF*	*61 h 28 min 12 s*	*6 min 14 s*
3DIRCADb	3D U-Net	31 h 59 min 35 s	13.75 s
	3D ResU-Net	32 h 02 min 30 s	14.23 s
	3D AI-UNet	33 h 34 min 29 s	14.67 s
	**3D DA-UNet**	**34 h 01 min 50 s**	**15.19 s**
	*3D DA-UNet* + *CRF*	*34 h 01 min 50 s*	*2 min 15 s*
SLiver07	3D U-Net	26 h 09 min 09 s	27.93 s
	3D ResU-Net	26 h 24 min 17 s	28.18 s
	3D AI-UNet	26 h 57 min 49 s	28.65 s
	**3D DA-UNet**	**27 h 59 min 37 s**	**29.02 s**
	*3D DA-UNet* + *CRF*	*27 h 59 min 37 s*	*3 min 25 s*

Significant values are in bold and italics.

### Comparisons

Table 4 compares the proposed methods in the 3DIRCADb test dataset with the deep learning-based SOTA methods. It can be seen from the table that our results are superior to other listed 2D-based methods on the five metrics. However, it is slightly inferior to the 3D H-DenseUNet on Dice and RVD proposed by Li et al.^11^.

**Table 4:** Comparisons with the SOTA methods on 3DIRCADb test datasets.

Model	Method	Dice (%)	VOE (%)	RVD (%)	ASD (mm)	RMSD (mm)
Christ et al.^3^	2D Cascaded FCN	90.23 ± 2.65	14.28 ± 4.58	− 2.55 ± 1.22	7.21 ± 3.95	10.22 ± 3.96
Chlebus et al.^30^	2D U-Net	93.36 ± 1.63	10.32 ± 3.12	− 1.19 ± 1.01	5.32 ± 3.01	7.45 ± 6.25
Han et al.^6^	2D ResNet	93.85 ± 1.25	9.55 ± 2.11	− 1.02 ± 0.98	5.98 ± 3.12	7.22 ± 7.12
Seo et al.^13^	2D mU-Net	96.25 ± 1.01	8.45 ± 2.02	0.97 ± 0.24	3.87 ± 1.21	6.25 ± 2.01
Li et al.^11^	3D H-DenseUNet	**98.74 ± 0.21**	7.47 ± 2.12	**0.16 ± 0.09**	1.22 ± 1.11	2.85 ± 3.11
Proposed	3D DA-UNet + CRF	98.17 ± 0.19	**3.58 ± 0.38**	0.18 ± 0.12	**0.95 ± 1.31**	**2.57 ± 0.32**

Significant values are in bold.

On the one hand, such results are due to the use of 3D convolution so that adjacent slices' spatial information is effectively used, and on the other hand, it benefits from the DS mechanism, which simultaneously establishes short-circuit connections and dense connections between the front and back layers of the network, and achieves feature reuse. Besides, the DS effectively solves the problem of gradient explosion/vanishment in model training. It makes the update process of the hidden layer filter more inclined to focus on high-resolution object features. Thus, to some extent, it is proved that the DS can make the model pay more attention to the target region.

Moreover, the general network output is the prediction with the maximum probability; there is no guarantee that each prediction is correct. However, the CRF has a transfer feature, which considers the order of output labels. The CRF layer can add some constraints to the last predicted labels to ensure that the predicted labels are legal, which can be learned automatically from the CRF layer.

**Figure 6:**
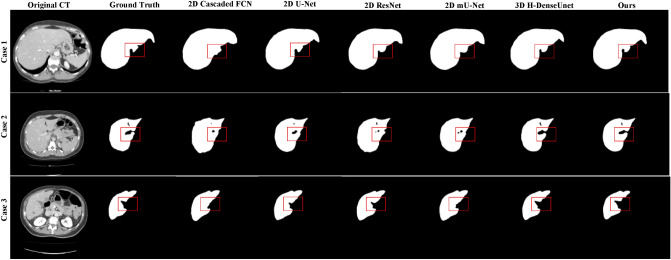
Three typical segmentation results of different SOTA methods on the 3DIRCADb test datasets.

Some typical segmentation results are illustrated in Fig. 6. As is shown, the results based on 2D methods showed more obvious errors (marked in red): in case 1 (small liver region), there is some under-segmentation. In case 2 and case 3 (blurred liver boundary), there is over-segmentation. These problems are mainly because the 2D method destroys the spatial information between slices of the original image, making each slice independent. However, the method proposed by Li et al.^11^ and ours fully considers the spatial information between slices to make the segmentation result closer to the ground truth.

The number of network parameters, training, and testing time, are listed in Table 5, among which the network with the fastest training is the network proposed by Han et al.^6^. It is based on CNN and borrows the skip connection and upsampling of U-Net, but does not use the exact number of convolution sequences as U-Net. Hence, the number of parameters is also the least. However, since it takes adjacent slice blocks as input, its segmentation accuracy is higher than Christ et al.^3^ and Chlebus et al.^30^. On the other hand, Li et al.^11^ took the most training time because they used a coarse-to-fine segmentation process, not only using 3D convolution but also 2D convolution in exchange for the most appropriate segmentation effect. However, the number of parameters of the method proposed in this paper is about 32 times lower than that of Li et al.^11^. Besides, the training and testing time is also significantly reduced, and the segmentation accuracy is very close to Li et al.^11^. Therefore, based on the above analysis, our proposed has comparable liver segmentation ability to the SOTA methods.

**Table 5:** Comparative results with the SOTA methods on parameters and time–cost on the 3DIRCADb dataset.

Model	Dimension	Parameters	Training time	Test time
Christ et al.^3^	2D	19,643,746	3 h 23 min 18 s	158.11 s
Chlebus et al.^30^	2D	7,765,442	2 h 45 min 33 s	139.85 s
Han et al.^6^	2D	**3,858,420**	**2 h 11 min 04 s**	**114.22 s**
Seo et al.^13^	2D	4,086,690	3 h 31 min 12 s	137.47 s
Li et al.^11^	3D	2,059,346,00	42 h 51 min 45 s	45.51 s
Proposed	3D	**6,422,037**	**34 h 01 min 50 s**	**15.19 s**
Proposed + CRF	–	–	*34 h 01 min 50 s*	*2 min 15 s*

Significant values are in bold and italics.

### Challenges

To evaluate the performance of the proposed method, we participated in the MICCIA-LiTS17 challenge and compared our proposed approach with other published deep learning-based methods. Table 6 lists the comparing results of the top-ranked SOTA methods (our team's name: HUSTWH402) (The result is publicly available at https://competitions.codalab.org/competitions/17094#results).

**Table 6:** Comparisons on LiTS17 challenge.

Method	Dimension	Model	DPC (%)	DG (%)
Roth et al.^10^	2D	U-Net	95.0	94.0
Kaluva et al.^7^		FCN	91.2	92.3
Liu et al.^33^		GIU-Net	–	95.05
Song et al.^31^		BS U-Net	96.1	96.4
Li et al.^11^	3D	H-DenseUNet	96.1	**96.5**
Jin et al.^12^		RA U-Net	**96.3**	96.1
Rafiei et al.^32^		U-Net	–	92.8
Ours		DA-UNet	95.3	95.8

Significant values are in bold.

It can be seen from Table 5 that our proposed method obtained 95.3% for Dice per case (DPC) and 95.8% for Dice global (DG) (ranking 16th and 13th, respectively). Although our result surpasses most D-based segmentation approaches, it is slightly lower than H-DenseUNet and Ra U-Net. As for the reason, the two 3D-based methods both employed 2D pre-training before formal 3D network processing. For example, Li et al.^11^ first used deep 2D DenseUNet for intra-slice feature extraction and then 3D H-DenseUNet for hybrid feature exploration. Similarly, Jin et al.^12^ used 2D input for liver localization (RA-UNet-I) and then used 3D input for liver segmentation (RA-UNet-II). Therefore, although their segmentation accuracy is improved, the end-to-end framework is destroyed to a certain extent.

### Advantages

**Figure 7:**
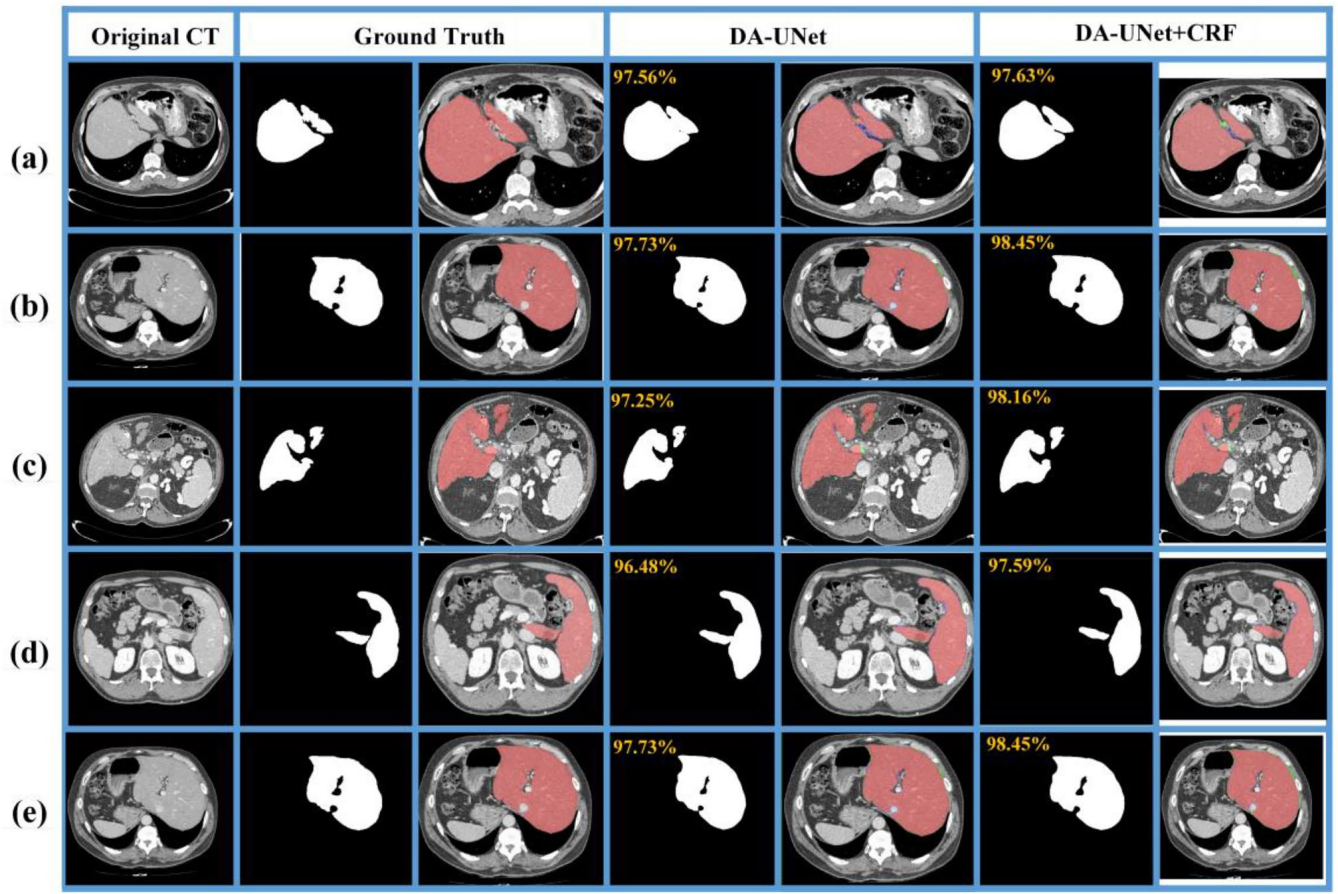
Visualization of segmentation error with the proposed 3D DA-UNet and post-processing on the LiTS17 dataset. (The red region denotes the ground truth, blue/green region represents the under-/over-segmentation. (**a**,**b**) liver with fuzzy boundaries, (**c**,**d**) liver with the obvious discontinuous regions, (**e**) liver with blood vessels inside.

This section illustrates some challenging cases using the proposed method. Figure 7a and b shows livers with fuzzy boundaries. As can be seen from the figures, the blurred edge connecting the liver region is segmented with slight error. Figure 7c and d shows a discontinuous liver with adjacent organs. It can be seen that the model shows a slight over-segmentation error. Figure 7e liver with blood vessels inside. There is a slight error around the vessel regions. However, after 3D dense CRF post-processing, the segmentation result is close to the ground truth.

The proposed model demonstrates superiority in handling challenging cases such as large and small liver regions, liver discontinuities, and livers containing blood vessels. The main reasons are as follows: Firstly, we upgrade the 2D convolution of U-Net to 3D convolution, making full use of the information between slices. Secondly, by adding residual connections between each convolution block, we make the network passes through a convolutional layer with a residual structure to extract more complex related features. Thirdly, the introduced DS mechanism into the decoding area makes the network focus on the relevant features of the shallow layer, which enable the top layer to output better discrimination and higher accuracy. Fourthly, using the Tversky loss function, by adjusting the parameters α and β, the model makes a good trade-off between FP and FN, effectively avoiding over-/under-segmentation. Finally, the 3D dense CRF is used as the post-processing to optimize the tiny boundaries further.

### Limitations

**Figure 8:**
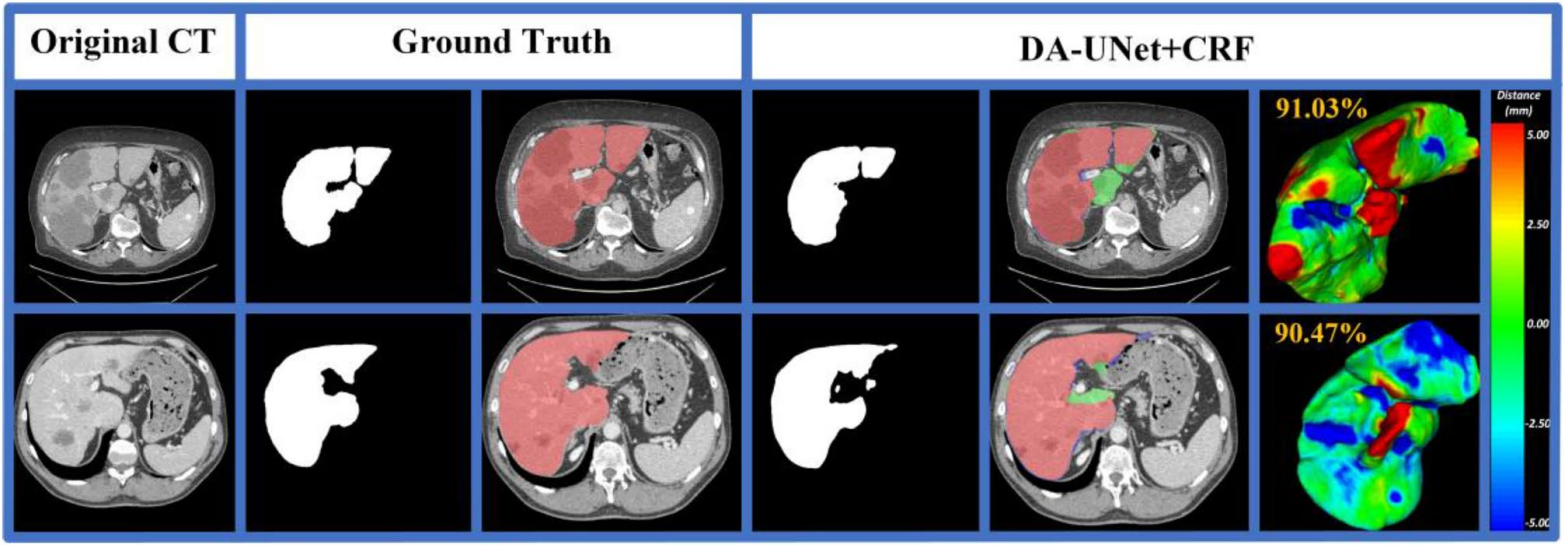
Illustrations of limitations of the proposed model with liver boundaries containing tumors. In the 2D error visualization, the red region denotes the ground truth, blue/green region represents the under-/over-segmentation. In the 3D error visualizations, the blue/red indicates under-/over-segmentation, and green indicates consistent results with the ground truth.

We illustrate some limitations on liver cases with neighboring organs of low contrast. When pathology liver tumors are at the boundary, our proposed method may result in significant over-/under-segmentation errors (Fig. 8). Therefore, the proposed model could achieve superior results when the liver contains low contrast with neighboring organs. However, it is still prone to errors when part of the liver border contains tumors.

## Conclusion

This paper proposes a novel liver segmentation framework based on U-Net, leveraging the DS, AI, and 3D dense CRF. The core idea of this method is to build a network that extracts deep image information together with full use of shallow features. First, we evaluated the proposed method on the datasets of LiTS17, 3DIRCADb, and SLiver07 using different hyperparameters of the Tversky loss function. Extensive experimental results show that when the hyperparameters α and β take 0.4/0.6, the segmentation effect is optimal. Moreover, we also compared the proposed DA-UNet with the SOTA methods and participated in the MICCIA-LiTS17 challenge. The result proved the comparative performance with the SOTA methods. Finally, we also demonstrated some typical difficult-to-segment cases on the three databases, which further confirmed our method's superiority for neighboring liver organs with low contrast.

In conclusion, the proposed method could achieve superior results in accurate liver segmentation. However, it still has certain limitations: (1) the amount of 3D network parameters is large, making the training process difficult and slow. (2) The proposed method is prone to errors when dealing with the liver with boundary tumors. Therefore, in future work, we aim to simplify the network architecture and alleviate the limitations mentioned above, such as adding a bottleneck layer to the network to reduce network parameters while maintaining high accuracy and effectiveness. Furthermore, since this paper aims at liver segmentation, it may not be suitable for direct application to more complex liver tumor segmentation scenarios. Therefore, we will pay more attention to liver tumor segmentation for a more comprehensive study and analysis in future work.

## Data Availability

The three datasets used in this paper are publicly available as follows: LiTS17 (https://competitions.codalab.org/competitions/17094), 3DIRCADb (https://www.ircad.fr/research/3d-ircadb-01/), and SLiver07(https://sliver07.grand-challenge.org/).
